# Supplementary material for: Genome-wide association analysis of left ventricular imaging-derived phenotypes identifies 72 risk loci and yields genetic insights into hypertrophic cardiomyopathy
Source: Nat Commun. 2023 Nov 30;14:7900. doi: 10.1038/s41467-023-43771-5 (PMC10689443; doi:10.1038/s41467-023-43771-5)
Supplement: Supplementary file 1 — Supplementary Information [file 41467_2023_43771_MOESM1_ESM.pdf]

Supplementary Information Content

Supplementary Methods

Supplementary Fig. 1. Sample selection workflow

Supplementary Fig. 2. Phenotypic correlation and PCA analysis of 12 LVRWT traits

Supplementary Fig. 3. Histogram of LVRWT phenotypes and residuals

Supplementary Fig. 4. Quantile-quantile plots of LVRWT GWAS *P* values

Supplementary Fig. 5. Heatmap of pleiotropic variants distribution of each LVRWTs

Supplementary Fig. 6. Annotation of candidate genes by using four different approaches

Supplementary Fig. 7. Heatmap of pleiotropic gene distribution of each LVRWTs using four methods

Supplementary Fig. 8. Distribution of PRS and cumulative incidence of HCM stratified by PRS of inferior LVRWT

Supplementary Fig. 9. Distribution of PRS and cumulative incidence of HCM stratified by PRS of inferolateral LVRWT

Supplementary Fig. 10. Distribution of PRS and cumulative incidence of HCM stratified by PRS of anterolateral LVRWT

Supplementary Fig. 11. Distribution of PRS and cumulative incidence of HCM stratified by PRS of anternor LVRWT

Supplementary Fig. 12. Distribution of PRS and cumulative incidence of HCM stratified by PRS of anterospetal LVRWT

Supplementary Fig. 13. C-statstic results of 12 LVRWT PRSs for HCM prediction

Supplementary Fig. 14. Graphical representation of GWAS on left ventricular regional wall thicknesses based on CMR imaging

Supplementary Table 1. Cohort characteristics of the UK Biobank

Supplementary Table 2. Cardiac magnetic resonance variables

Supplementary Methods

Deep learning model performance

We used the Automated Cardiac Diagnosis Challenge (ACDC) dataset to train a deep learning-based segmentation model. ACDC took place during the MICCAI 2017 conference and the dataset of ACDC was used for extensive research[1,2]. The ACDC dataset contains short-axis cine-MRI images from real clinical exams at the University Hospital of Dijon. The images were taken from 100 subjects from four pathological groups and one healthy group: patients with previous myocardial infarction, dilated cardiomyopathy, hypertrophic cardiomyopathy and abnormal right ventricle, as well as normal subjects. The images range temporally from 28 to 40 frames covering completely or partially the cardiac cycle, and spatially from the base to the apex. Masks of LV cavity, LV myocardium were annotated for the end-diastolic (ED) and end-systolic (ES) frames by one clinical expert. In total, the dataset contains 1902 annotated images. 1420 short axis images were employed for training the deep learning model, 100 short axis images for validating and selecting the model with best performance. Within the total 382 testing images, 80 images belong to the mid-cavity slices that were ultimately used to test model performance. To demonstrate the DLANet have ability to perform well also on the UKB dataset, we resorted to a doctor who has 7 years of clinical experience to label the LV cavity and LV myocardium. A total of 50 patients were annotated, with 5 randomly selected frames for each patient, and in 8-14 slices per frame. Among these annotations, we focused on selecting the mid-cavity slices to form the UKB testing set, and result in total of 500 short-axis images.

To score the quality of manual and automated segmentations, prediction results of 382 testing images were compared to the ground-truth labels using the Dice score[3,4] and Hausdorff Distance (HD)[5]. The Dice score scales from 0 (no agreement between manual and automated annotations) to 1 (perfect agreement) and the HD is generally defined as the longest distance between any point on one set and its closest neighbor in the other set and thus provides a good measure for the maximum distance between two segmentations.

We compared the DLANet with state-of-the-art methods by re-implementing them and reporting the results on different datasets in **Supplementary Data 21** and **Supplementary Data 22**. In Supplementary Data 21, DLANet achieved the best segmentation results in terms of both Dice and HD metrics. Additionally, for the UKB dataset, all testing results surpassed those of the ACDC dataset due to the majority of UKB cases being healthy, making the task of segmentation easier. Supplementary Data 22 shows that DLANet also achieved a good overall performance with an average Dice of 0.962 and an average HD of 2.051 mm. More segmentation results on UKB dataset are shown in **Supplementary Fig 15**. Prediction results generated by the DLANet have a good consistency with the manual labels by the doctors.

Measurement of left ventricular regional wall thickness from the short axis view

For quantification, we designed a measurement-based method to calculate wall thicknesses. Before the measurement, we normalize the MR image[6] by rotation to the fixed arrangement in order to perform subsequent measurements. The thicknesses rely on the calculation of the distance between the center and the contour of the mask, either epicardium or endocardium, for some direction. The center can be calculated as the geometric center of the mask, and the contour is the collection of the outermost pixels of the mask. However, even presented with the center and contour, it was not trivial to measure the distance in the discretized images. To reduce the possible errors introduced by the discretization, we assumed the distance is an underlying function of the direction angle, and approximate the function with key points in the contour. Specifically, we first located the key points $\{(x_k, y_k)\}_{k=1}^{K}$ of the contour, and transform the Cartesian representation to the polar representation $\{(r_k, \theta_k)\}_{k=1}^{K}$ with the center $C(x_0, y_0)$ as the pole. The coordinates $\{(r_k, \theta_k)\}_{k=1}^{K}$ of the key points were then used to approximate the function $f: \theta \rightarrow r$ with 1-D interpolation, and finally, we evaluated the function f at desired direction θ to get the corresponding distance $r = f(\theta)$. This function approximation procedured takes advantage of the smoothness of the contour, and thus was a better way to handle the discretization issue, compared with directly measuring the distance. With the procedure, the thicknesses were readily derived: the difference of distances for the same direction, with one distance between center and endocardium contour and the other between center and epicardium contour. Furthermore, considering the possible noise in the segmentation mask, and measuring in a single direction θ may not be reliable, as a result, we measured in several uniformly spaced direction angles $\{\theta_t | \theta_t \in [\theta - \delta\theta, \theta + \delta\theta], t = 1,2,\cdots,T\}$ in the neighborhood $[\theta - \delta\theta, \theta + \delta\theta]$ and took an average of the resulting distances for a robust quantification:

$$r = \frac{1}{T}\sum_{t=1}^{T} f(\theta_t)$$

We averaged ten measurements for wall thicknesses. We used the trained segmentation model and measurement method to calculate the myocardial thicknesses on the UKB dataset, which contains over 50,000 subjects. The generated results were employed for the following analyses.

Evaluation for measurement of left ventricular regional wall thickness

For the evaluation metric, we employed Mean Absolute Error (MAE) and it is defined as:

$$MAE = \frac{1}{N}\sum_{i=1}^{N} |\hat{y}_i - y_i|$$

Where y_i and $\hat{y}_i$ denotes the label and prediction of wall thickness for image i.

From Supplementary Data 21 and Supplementary Data 22, all methods achieved accurate quantification results, with the MAE of LVRWT are at most of 0.987 mm for the ACDC dataset and 0.745 mm for the UKB dataset. We would like to emphasize that these values are comparable to the spacing represented by one pixel (ranging from 0.70 mm/pixel to 1.92

mm/pixel), and as a result demonstrate the models' reliability on clinical practice. The MSMM achieved the best MAE of 0.853±0.304 for ACDC and the second-best MAE of 0.663±0.343 for UKB.

To observe the sensitivity of the MAE of myocardial thickness, we tested the DLANet on both the ACDC and UKB datasets with different numbers of training images, i.e. 25%, 50%, 75%, and 100% of the training data. As shown **in Supplementary Data 23** and **Supplementary Data 24**, even when training the DLANet with only 25% of the images, the MAE of these two dataset are only at 0.945 mm and 0.807mm. While there are some improvements as the amount of training data increased, the differences between models trained with 25% and 100% of the data are not significant. These findings have two implications. On one hand, they demonstrate that the DLANet has the potential to generate accurate results even with a small number of input images. On the other hand, the mid-cavity images of the heart have high quality and strong contrast between the LV's endocardium and epicardium, which makes segmentation and quantification easier compared to slices located elsewhere.

Genomic annotation for risk variants of 12 LVRWT traits

For enrichment analysis, we first generated a set of control variants with the allele frequencies, number of variants in LD, as well as genomic distribution matched to risk variants using a web tool vSampler (http://mulinlab.org/vsampler/). SnpEff was used to annotate the genomic annotation for both risk variants and control variants. Variants would be classified into the following categories: upstream gene, downstream gene, 5'UTR, 3'UTR, intron, exon, intergenic region. Enrichment analyses of genomic annotation were performed by two-tailed Fisher's exact test with Bonferroni correction as the following 2 × 2 table (columns; risk variants and control variants rows; variants within and not within the annotated genomic region).

Enrichment analyses for risk variants of 12 LVRWT traits among functional annotation

The functional annotation files for ChIP-seq peaks of histone modification including H3K4me1, H3K4me3, H3K27ac, H3K36me3, H3K9me3 and transcriptional factor binding sites were downloaded from the ENCODE portal (https://www.encodeproject.org). BEDtools was used to identify risk variants or control variants overlapped with the peaks of regulatory elements. Enrichment analyses for risk variants of 12 LVRWT traits among regulatory elements were performed by two-tailed Fisher's exact test.

Enrichment analyses for risk variants of 12 LVRWT traits among CVDs-related GWAS loci

Data sources of summary statistics GWAS from 11 selected CVDs traits for enrichment correlation analyses were listed in **Supplementary Data 2.** BEDtools was used to examine whether risk variants or control variants located in GWAS loci. Enrichment analyses for risk variants of 12 LVRWT traits among CVDs-related GWAS loci were performed by two-tailed

Fisher’s exact test.

Supplementary Figure

Supplementary Fig 1

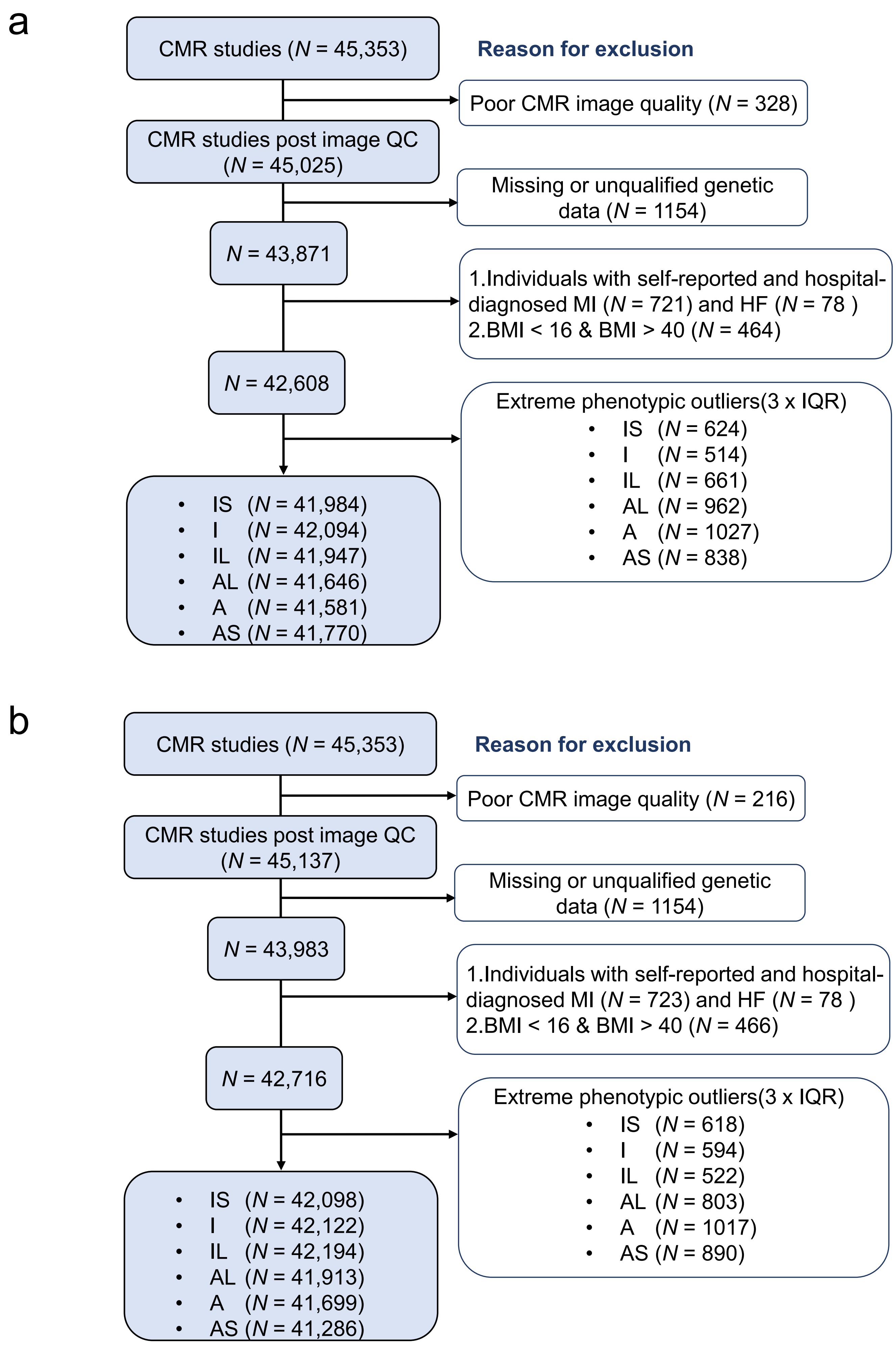

Supplementary Fig. 1. Sample selection workflow.

Sample selection flowchart for end-systole (**a**) and end-diastole (**b**). Abbreviation: CMR, cardiovascular magnetic resonance; IS, inferoseptal; I, inferior; IL, inferolateral; AL, anterolateral; A, anternor; AS, anterospetal; MI, myocardial infarction; HF, heart failure.

Supplementary Fig 2

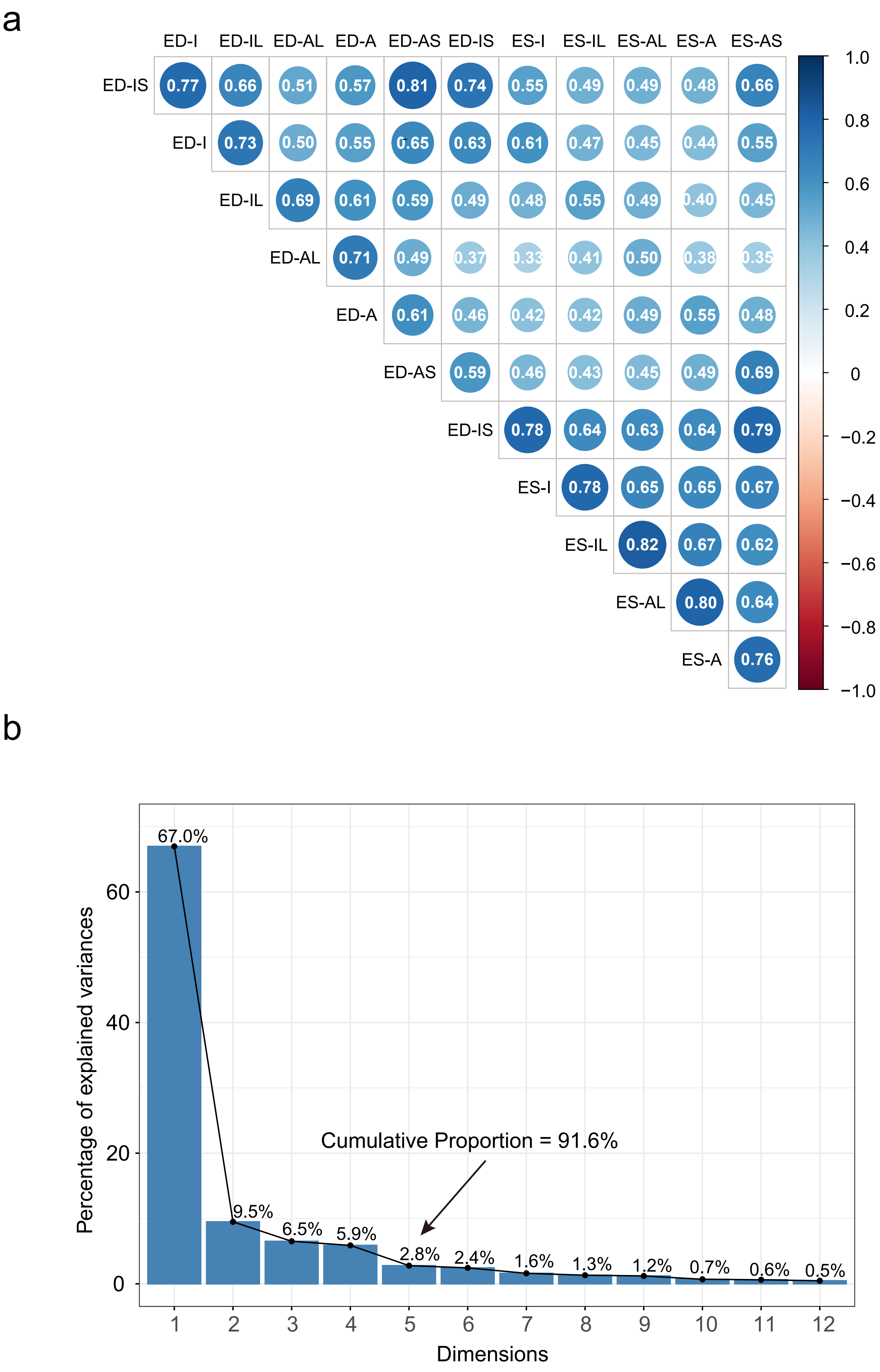

Supplementary Fig. 2. Phenotypic correlation and PCA analysis of 12 LVRWT traits.

a. Pearson correlations between 6 end-systole LVRWT phenotypes and 6 end-diastole LVRWT phenotypes. Degree of correlation is indicated by color legend and circle area, ranging from -1 to +1. Only significant correlations ($P < 0.05$, two-sided) are shown. **b.** the results of Principal Component Analysis (PCA) applied to the 12 LVRWT traits. Through PCA, we projected the original traits onto 5 principal components, which collectively explain 91.6% of the total variance. Abbreviation: LVRWT, LV regional wall thickness; MSMM, Myocardial Segmentation and Measurement Method; IS, inferoseptal; I, inferior; IL, inferolateral; AL, anterolateral; A, anternor; AS, anterospetal; ES, end systole; ED, end diastole.

Supplementary Fig 3

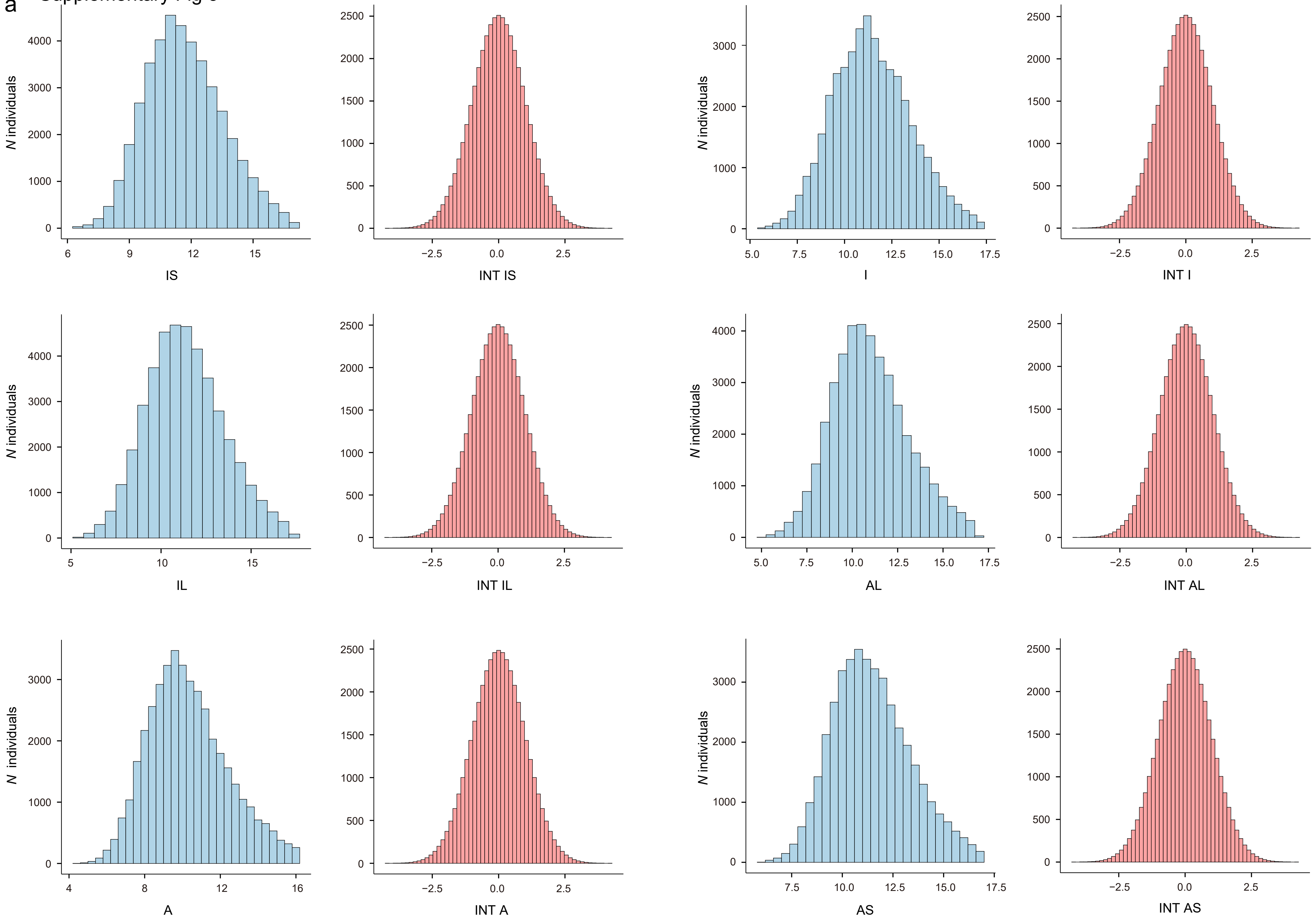

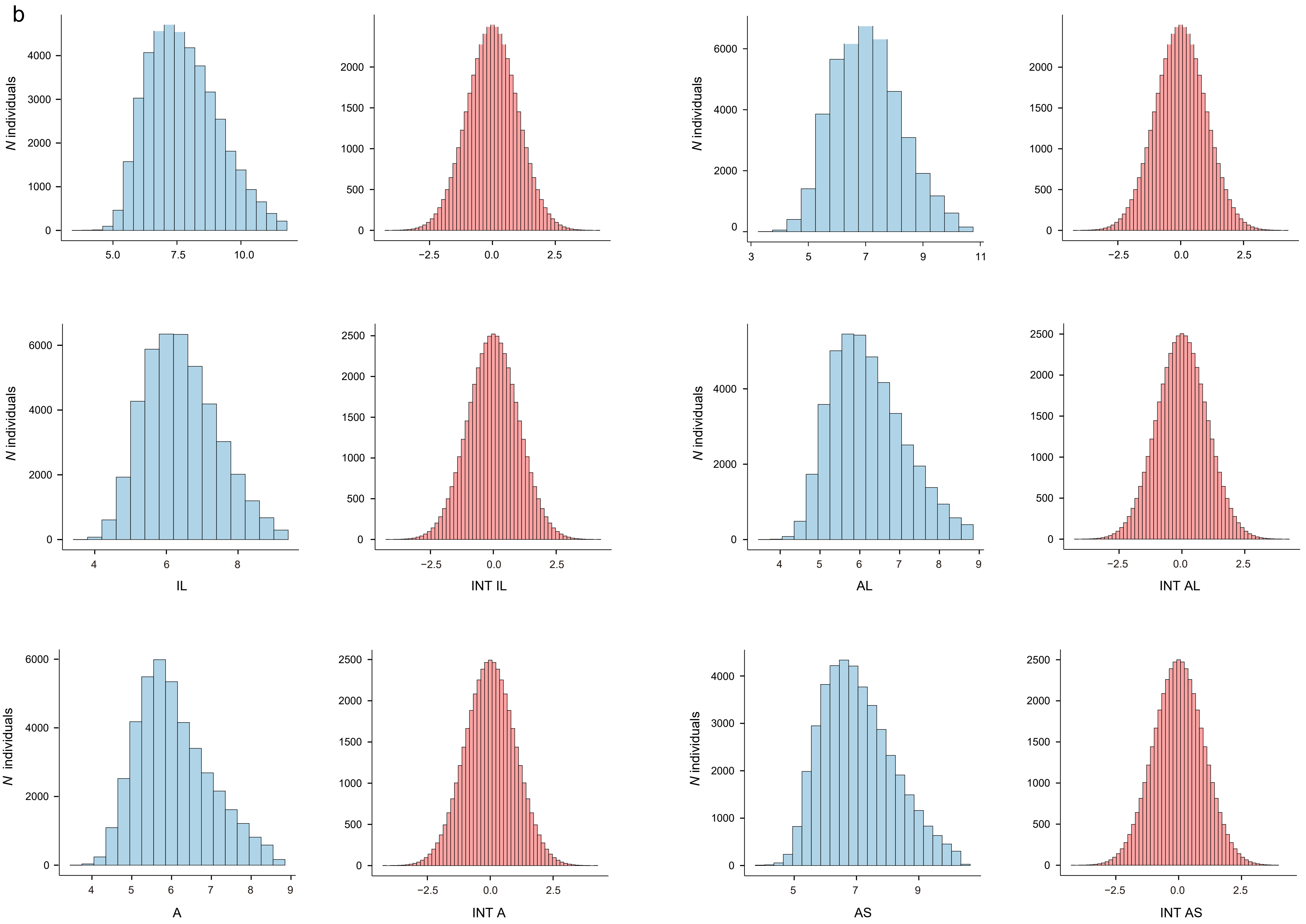
b
N individuals
IL
INT IL
AL
INT AL
A
INT A
AS
INT AS

Supplementary Fig. 3. Histogram of LVRWT phenotypes and residuals.

Histogram of LVRWT phenotypes for end-systole (**a**) and end-diastole (**b**). The blue histograms show the distribution of LVRWT phenotypes. The red histograms show the distribution of residuals which were rank-based inverse normal transformed after regressing against the variables (including sex, age, body mass index, imaging centre). Abbreviation: LVRWT, LV regional wall thickness; IS, inferoseptal; I, inferior; IL, inferolateral; AL, anterolateral; A, anternor; AS, anterospetal.

Supplementary Fig 4

a

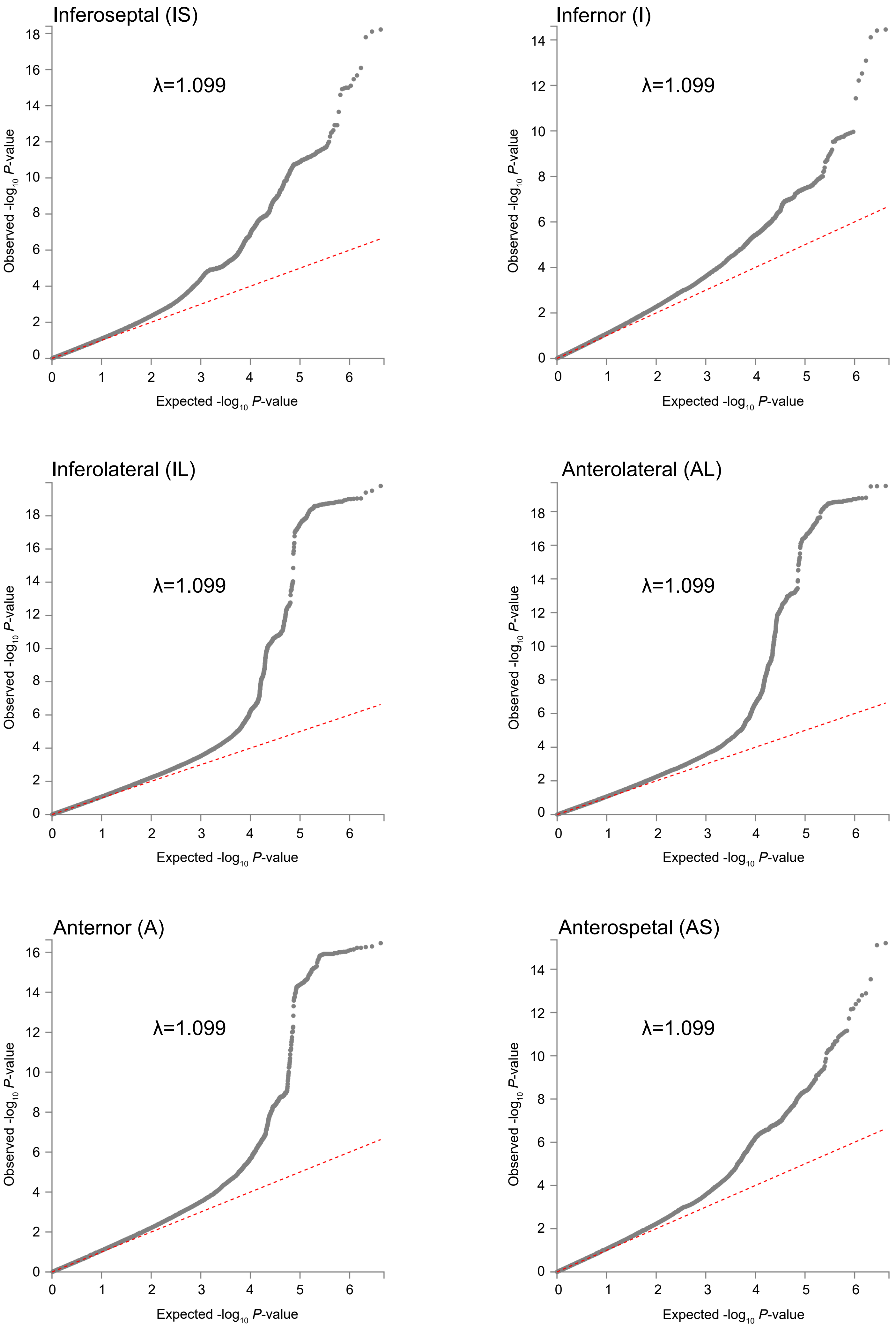

b

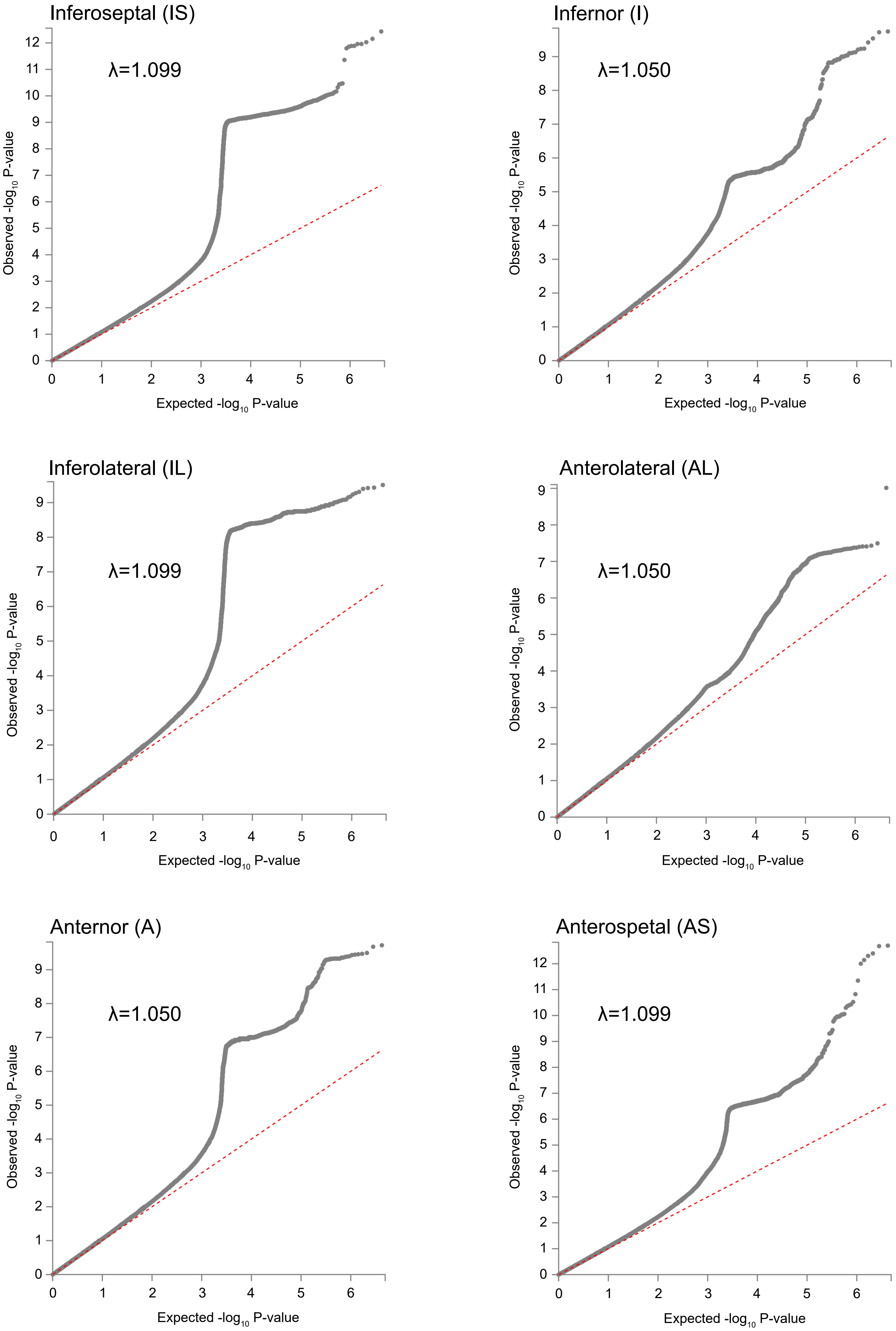

Inferoseptal (IS)
λ=1.099
Infernor (I)
λ=1.050
Inferolateral (IL)
λ=1.099
Anterolateral (AL)
λ=1.050
Anternor (A)
λ=1.050
Anterospetal (AS)
λ=1.099
Observed -log10 P-value
Expected -log10 P-value

Supplementary Fig. 4. Quantile-quantile plots of LVRWT GWAS *P* values.

Quantile-quantile plots of LVRWT for end-systole (**a**) and end-diastole (**b**). Quantile-quantile plots from GWAS of LVRWT traits show observed -log10(P) on Y-axis and expected -log10(P) on X-axis. λ, genomic inflation factor. Abbreviation: LVRWT, LV regional wall thickness; IS, inferoseptal; I, inferior; IL, inferolateral; AL, anterolateral; A, anternor; AS, anterospetal.

Supplementary Fig 5

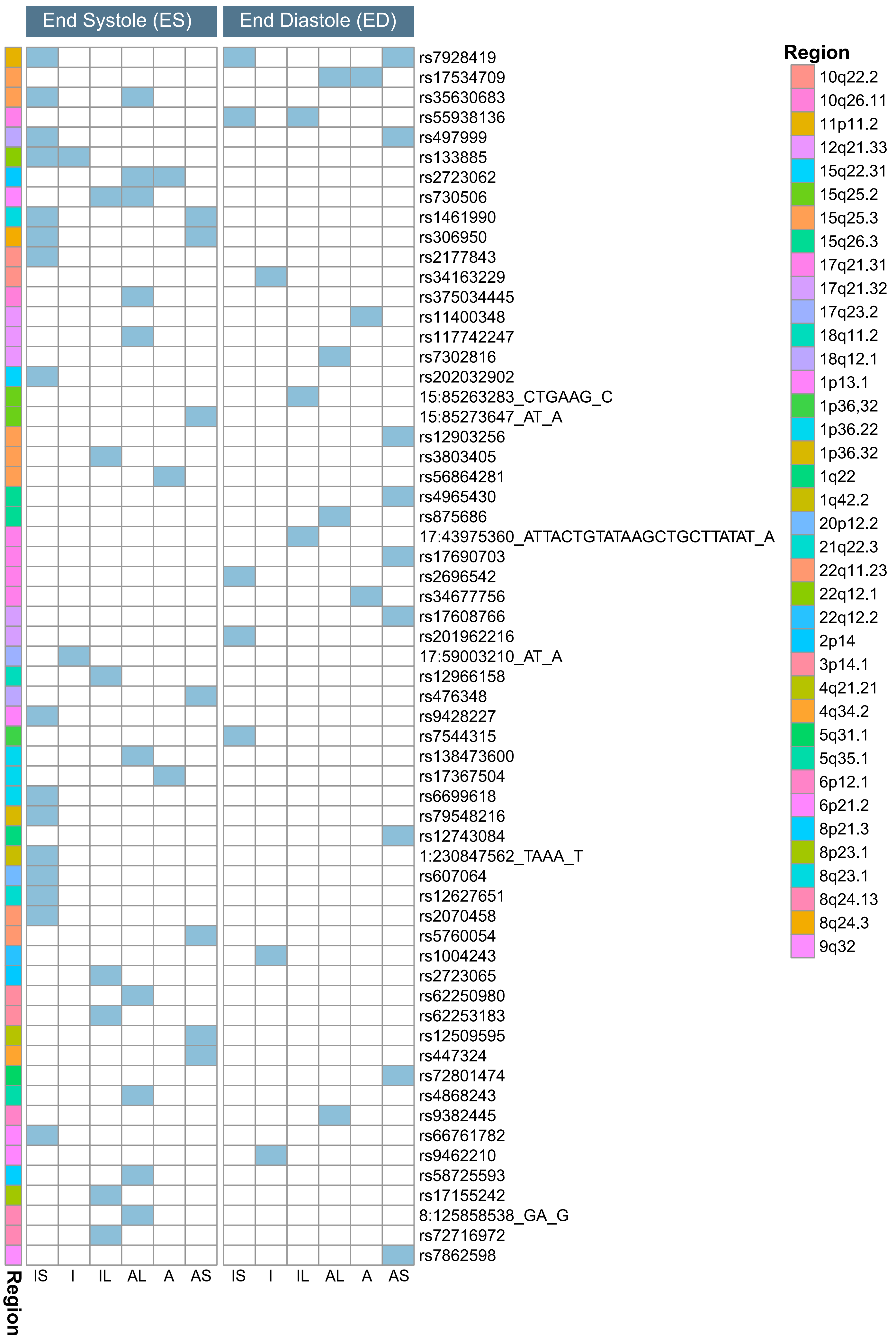

Supplementary Fig. 5. Heatmap of pleiotropic variants distribution of each LVRWTs

Abbreviation: LVRWT, LV regional wall thickness; IS, inferoseptal; I, inferior; IL, inferolateral; AL, anterolateral; A, anternor; AS, anterospetal; ES, end systole; ED, end diastole.

Supplementary Fig 6

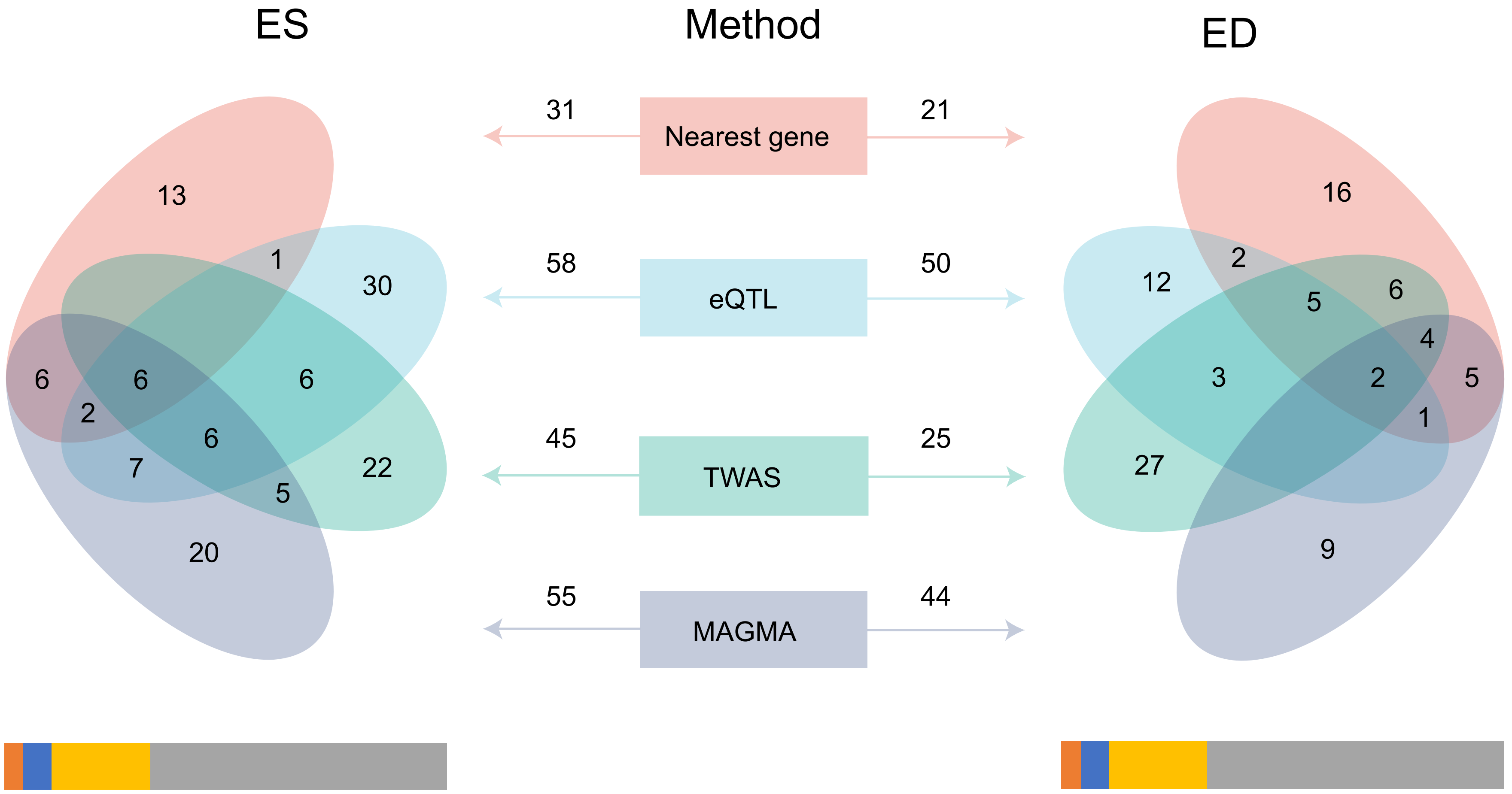

Supplementary Fig. 6. Annotation of candidate genes by using four different approaches.

The venn diagram at the upper left corner displays the candidate genes overlap between the four methods (Nearest gene, eQTL, TWAS, MAGMA) at the end-systole. The bar at the bottom left corner represents the proportion of genes annotated using the four methods at the end-systole: the orange represents the genes are annotated by four methods, the blue represents the genes are annotated by three methods, the yellow represents the genes are annotated by two methods, while the grey represents the genes are only annotated by one method. The venn diagram in the upper right corner displays the candidate genes overlap between the four methods (Nearest gene, eQTL, TWAS, MAGMA) at the end-systole. The bar in the bottom right corner represents the proportion of genes annotated using the four methods at the end-systole: the orange represents the genes are annotated by four methods, the blue represents the genes are annotated by three methods, the yellow represents the genes are annotated by two methods, while the grey represents the genes are only annotated by one method. Abbreviation: eQTL, expression quantitative trait locus; TWAS, transcriptome-wide analysis; MAGMA, Multi-marker Analysis of GenoMic Annotation.

Supplementary Fig 7

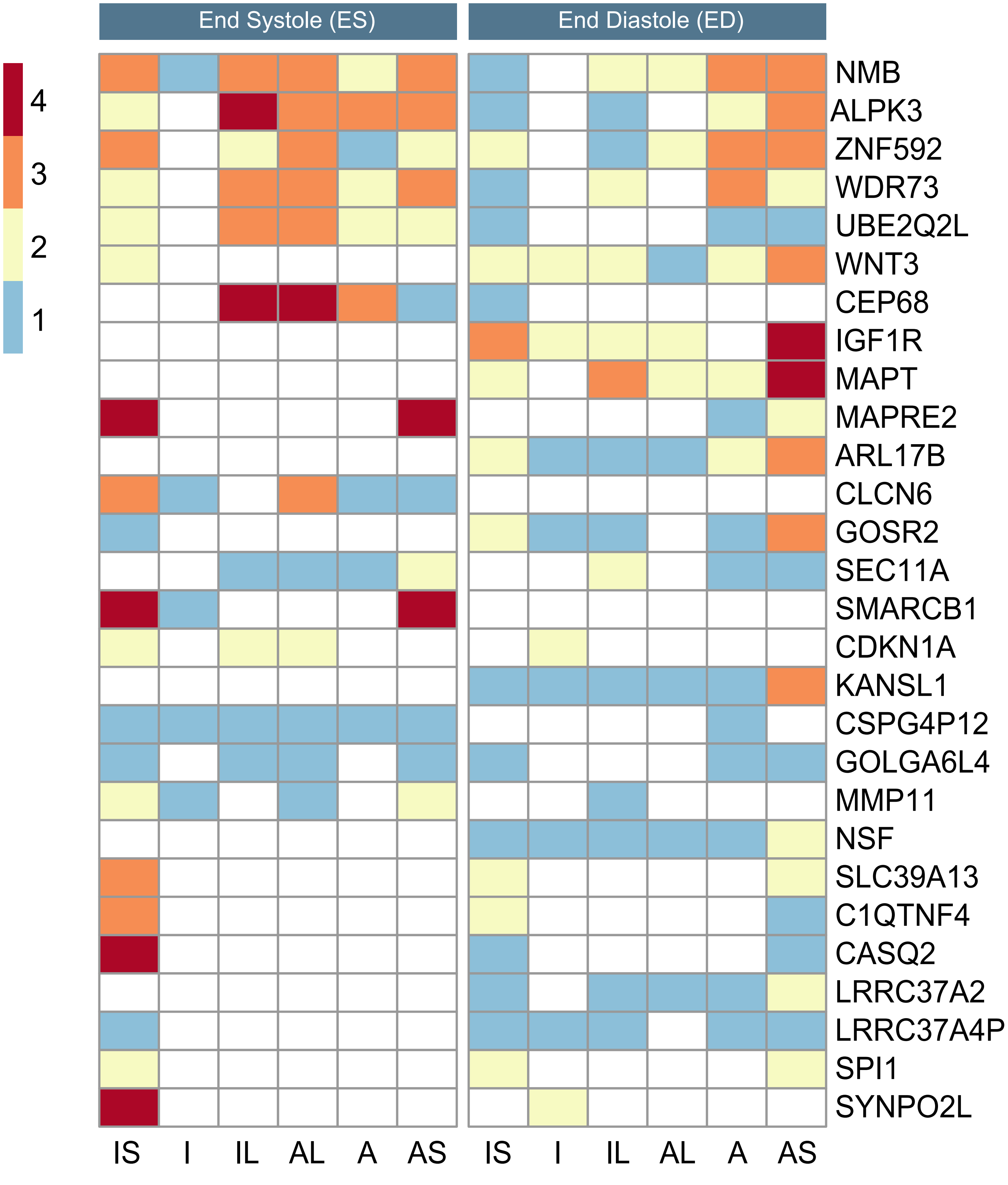

Supplementary Fig. 7. Heatmap of pleiotropic gene distribution of each LVRWTs using four methods.

The times of the gene annotated by four methods (Nearest Gene, eQTL, MAGMA, TWAS) are indicated by the color legend. For example, the ALPK3 gene can be annotated by the four methods in end-diastole inferior LVRWT. The maximum number of each cell is 4. Further details can see in Supplementary Table 12. Abbreviation: LVRWT, LV regional wall thickness; IS, inferoseptal; I, inferior; IL, inferolateral; AL, anterolateral; A, anternor; AS, anterospetal; ES, end systole; ED, end diastole.

Supplementary Fig 8

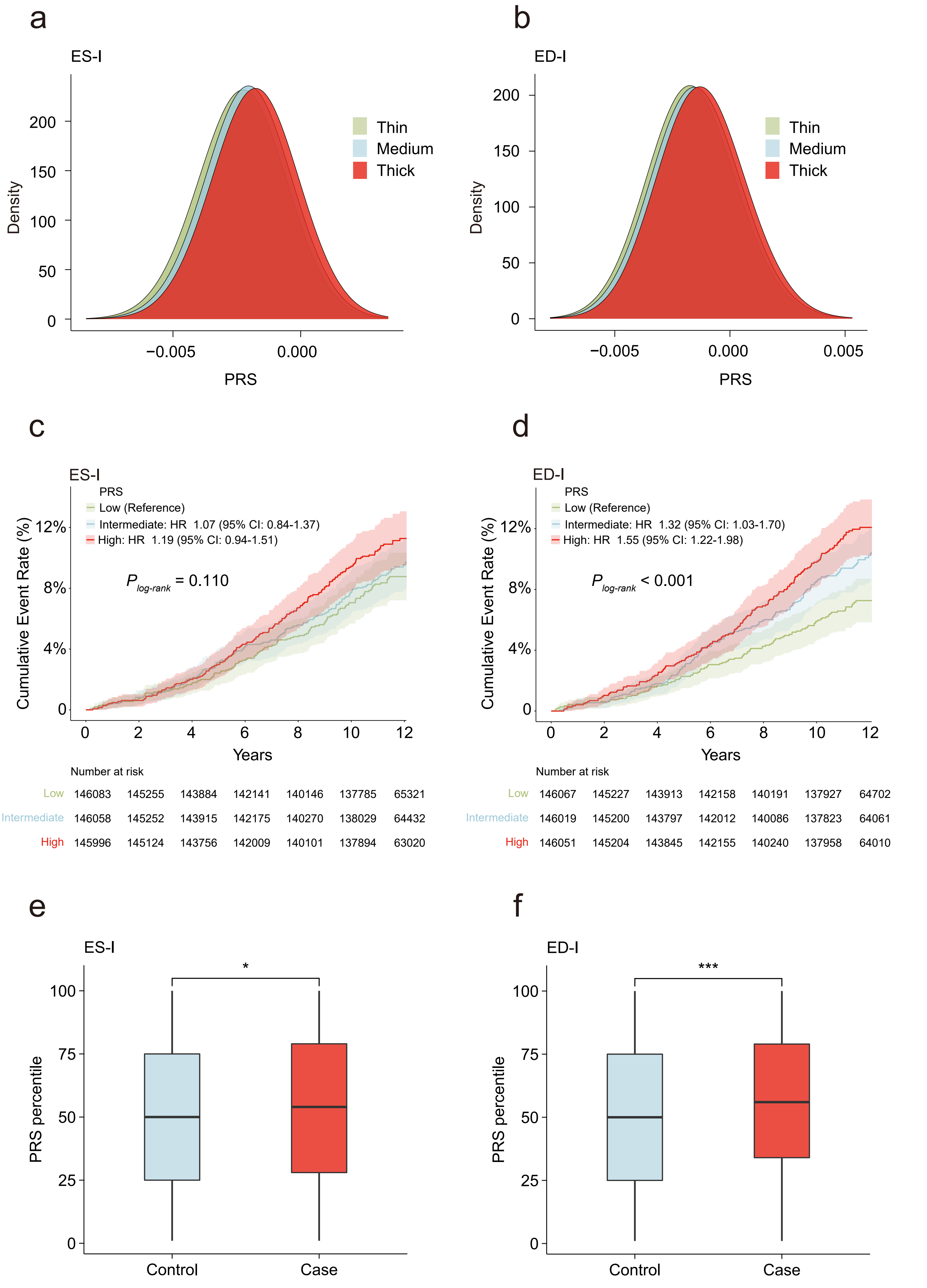

Supplementary Fig. 8. Distribution of PRS and cumulative incidence of HCM stratified by PRS of inferior LVRWT.

a-b. Distribution of PRS for participants with thin, medium and thick LVRWT. PRS of inferior LVRWT end systole (**a**) and end diastole (**b**) yielded discrimination for the LVRWTs. **c-d.** A total of 439,981 individuals unrelated to the CMR cohort. Those in the first tertiles of genetically predicted inferior LVRWT are depicted in green, the second tertiles are depicted in blue and the last tertiles are depicted in red. The darker shades represent the central estimate of the cumulative incidence (defined as 1-the Kaplan-Meier survival estimate). The lighter shades represent the respective 95% CIs. The x axis depicts years since enrollment in the UKB; the y axis depicts cumulative incidence of HCM. Strata based on genetic prediction of inferior LVRWT at end systole (**c**) and end diastole (**d**). **e-f.** Distribution of ES-I (**e**) and ED-I (**f**) PRS percentiles for HCM cases (n = 420) and controls (n = 439,561). For all box plots: central line of each box, median; top and bottom edges of each box, first and third quartiles; whiskers extend 1.5× the interquartile range beyond box edges. *P*-values were calculated by a two-sided Student's t-test. Asterisk denotes statistically significant differences * *P* <0.05; ** *P* <0.01; *** *P* <0.001. Abbreviation: PRS, polygenic risk score; HCM, hypertrophic cardiomyopathy; LVRWT, LV regional wall thickness; ES, end systole; ED, end diastole; I, inferior.

Supplementary Fig 9

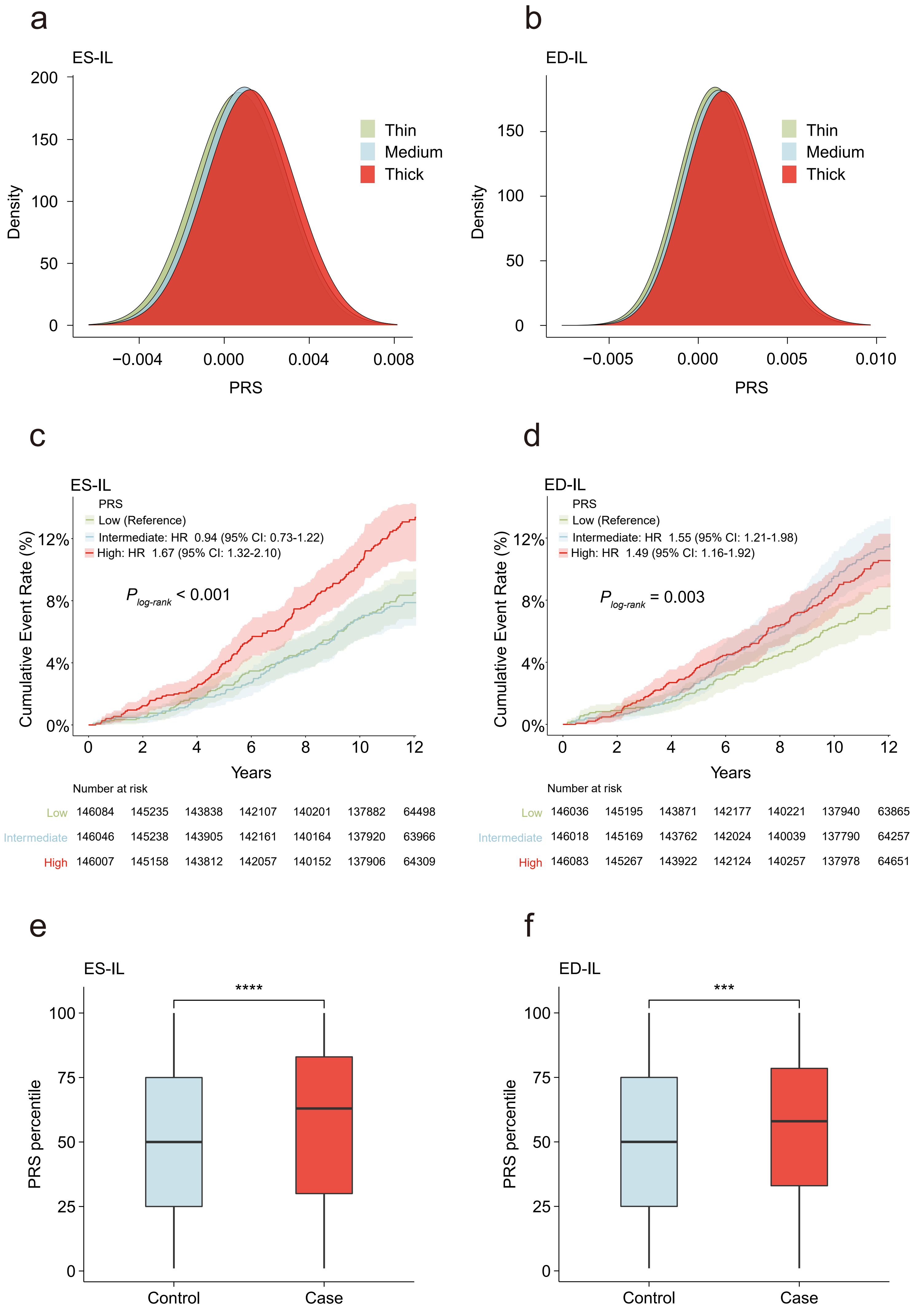

Supplementary Fig. 9. Distribution of PRS and cumulative incidence of HCM stratified by PRS of inferolateral LVRWT.

a-b. Distribution of PRS for participants with thin, medium and thick LVRWT. PRS of inferolateral LVRWT end systole (**a**) and end diastole (**b**) yielded discrimination for the LVRWTs. **c-d.** A total of 439,981 individuals unrelated to the CMR cohort. Those in the first tertiles of genetically predicted inferolateral LVRWT are depicted in green, the second tertiles are depicted in blue and the last tertiles are depicted in red. The darker shades represent the central estimate of the cumulative incidence (defined as 1-the Kaplan-Meier survival estimate). The lighter shades represent the respective 95% CIs. The x axis depicts years since enrollment in the UKB; the y axis depicts cumulative incidence of HCM. Strata based on genetic prediction of inferolateral LVRWT at end systole (**c**) and end diastole (**d**). **e-f.** Distribution of ES-IL (**e**) and ED-IL (**f**) PRS percentiles for HCM cases (n = 420) and controls (n = 439,561). For all box plots: central line of each box, median; top and bottom edges of each box, first and third quartiles; whiskers extend 1.5× the interquartile range beyond box edges. *P*-values were calculated by a two-sided Student's t-test. Asterisk denotes statistically significant differences * $P < 0.05$; ** $P < 0.01$; *** $P < 0.001$. Abbreviation: PRS, polygenic risk score; HCM, hypertrophic cardiomyopathy; LVRWT, LV regional wall thickness; ES, end systole; ED, end diastole; IL, inferolateral.

Supplementary Fig 10

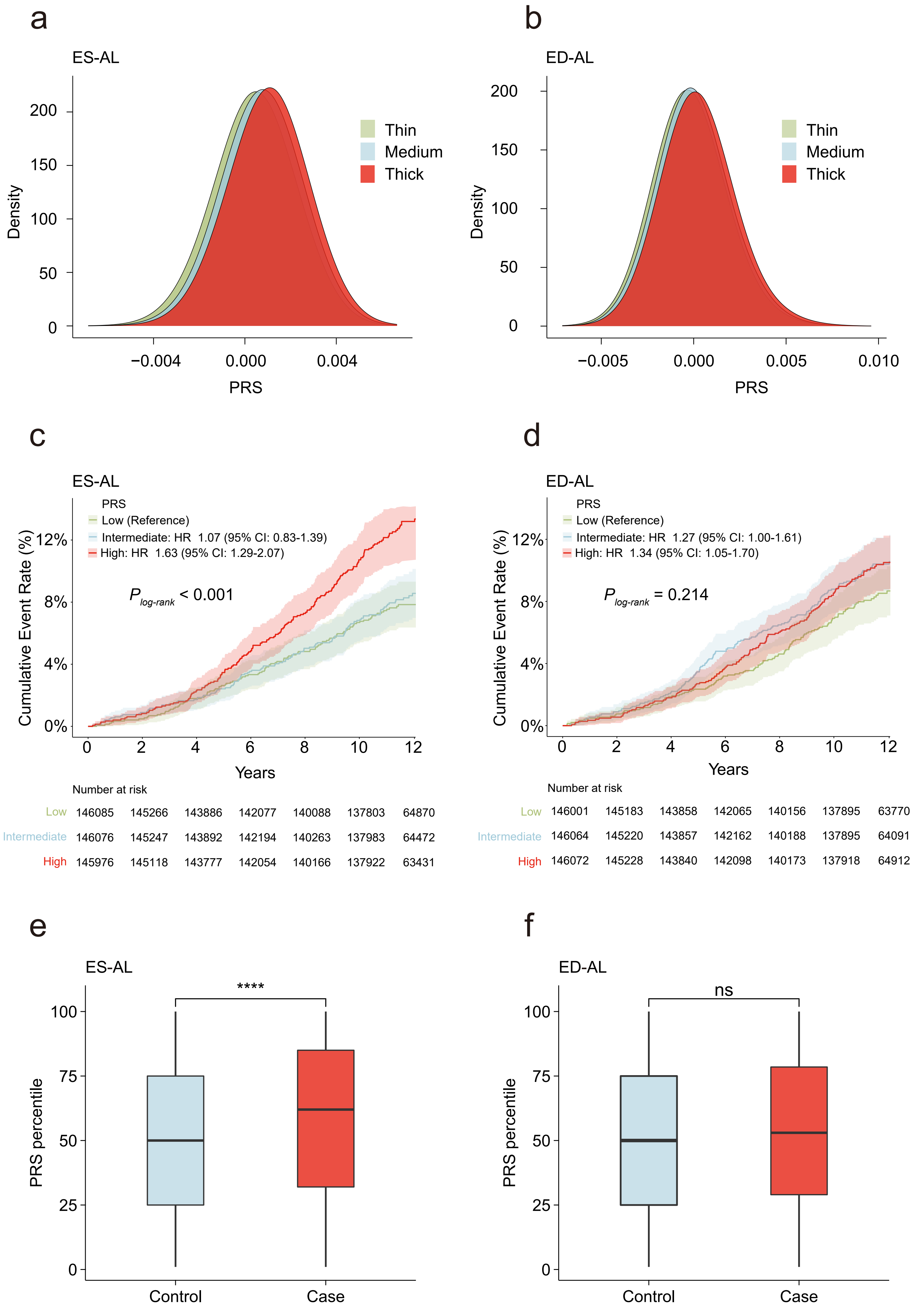

Supplementary Fig. 10. Distribution of PRS and cumulative incidence of HCM stratified by PRS of anterolateral LVRWT.

a-b. Distribution of PRS for participants with thin, medium and thick LVRWT. PRS of anterolateral LVRWT end systole (**a**) and end diastole (**b**) yielded discrimination for the LVRWTs. **c-d.** A total of 439,981 individuals unrelated to the CMR cohort. Those in the first tertiles of genetically predicted anterolateral LVRWT are depicted in green, the second tertiles are depicted in blue and the last tertiles are depicted in red. The darker shades represent the central estimate of the cumulative incidence (defined as 1-the Kaplan-Meier survival estimate). The lighter shades represent the respective 95% CIs. The x axis depicts years since enrollment in the UKB; the y axis depicts cumulative incidence of HCM. Strata based on genetic prediction of anterolateral LVRWT at end systole (**c**) and end diastole (**d**). **e-f.** Distribution of ES-AL (**e**) and ED-AL (**f**) PRS percentiles for HCM cases (n = 420) and controls (n = 439,561). For all box plots: central line of each box, median; top and bottom edges of each box, first and third quartiles; whiskers extend 1.5× the interquartile range beyond box edges. *P*-values were calculated by a two-sided Student's t-test. Asterisk denotes statistically significant differences * $P < 0.05$; ** $P < 0.01$; *** $P < 0.001$. Abbreviation: PRS, polygenic risk score; HCM, hypertrophic cardiomyopathy; LVRWT, LV regional wall thickness; ES, end systole; ED, end diastole; AL, anterolateral.

Supplementary Fig 11

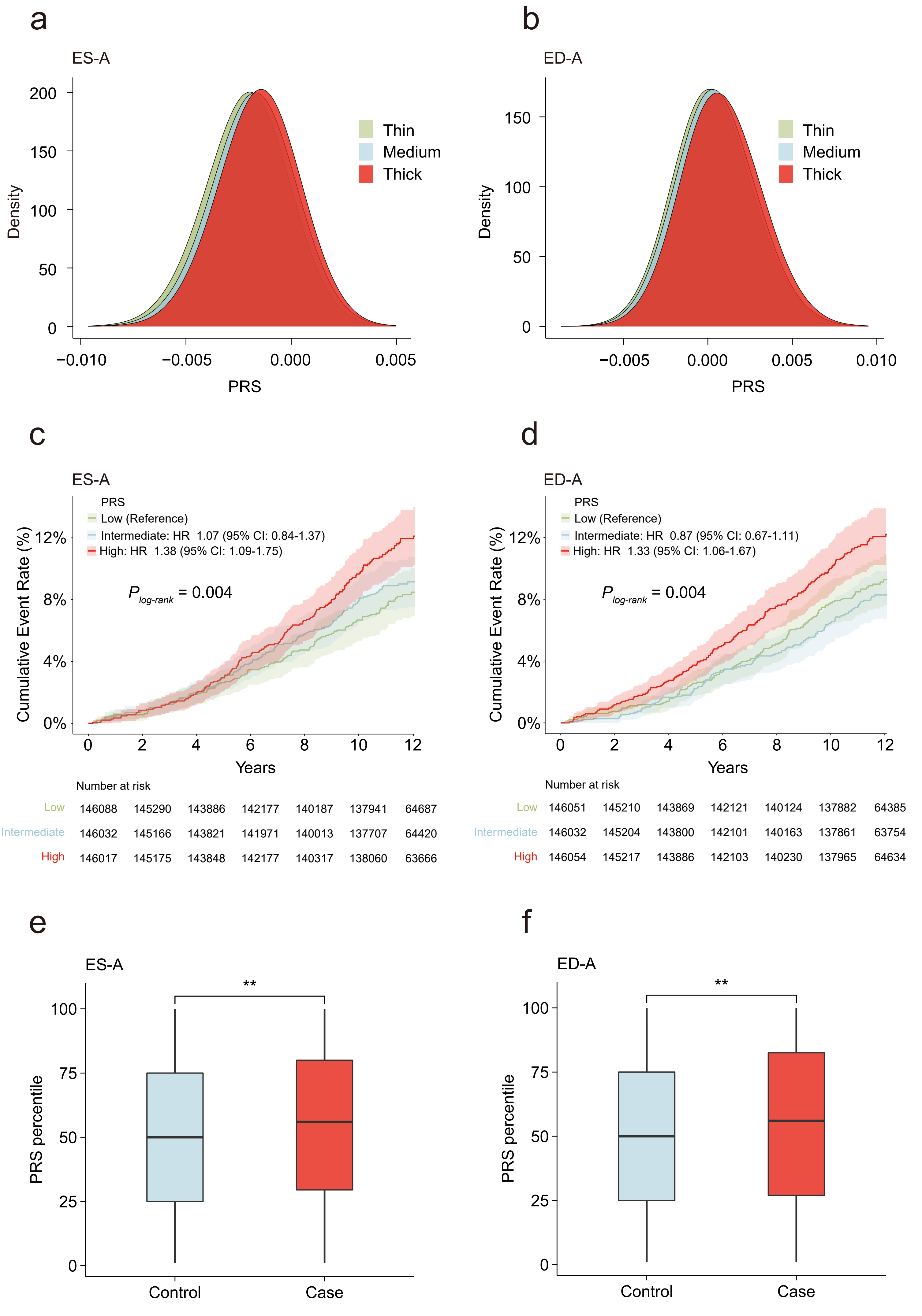

Supplementary Fig. 11. Distribution of PRS and cumulative incidence of HCM stratified by PRS of anternor LVRWT.

a-b. Distribution of PRS for participants with thin, medium and thick LVRWT. PRS of anternor LVRWT end systole (**a**) and end diastole (**b**) yielded discrimination for the LVRWTs. **c-d.** A total of 439,981 individuals unrelated to the CMR cohort. Those in the first tertiles of genetically predicted anternor LVRWT are depicted in green, the second tertiles are depicted in blue and the last tertiles are depicted in red. The darker shades represent the central estimate of the cumulative incidence (defined as 1-the Kaplan-Meier survival estimate). The lighter shades represent the respective 95% CIs. The x axis depicts years since enrollment in the UKB; the y axis depicts cumulative incidence of HCM. Strata based on genetic prediction of anternor LVRWT at end systole (**c**) and end diastole (**d**). **e-f.** Distribution of ES-A (**e**) and ED-A (**f**) PRS percentiles for HCM cases (n = 420) and controls (n = 439,561). For all box plots: central line of each box, median; top and bottom edges of each box, first and third quartiles; whiskers extend 1.5× the interquartile range beyond box edges. *P*-values were calculated by a two-sided Student's t-test. Asterisk denotes statistically significant differences * $P<0.05$; ** $P<0.01$; *** $P<0.001$. Abbreviation: PRS, polygenic risk score; HCM, hypertrophic cardiomyopathy; LVRWT, LV regional wall thickness; ES, end systole; ED, end diastole; A, anternor.

Supplementary Fig 12

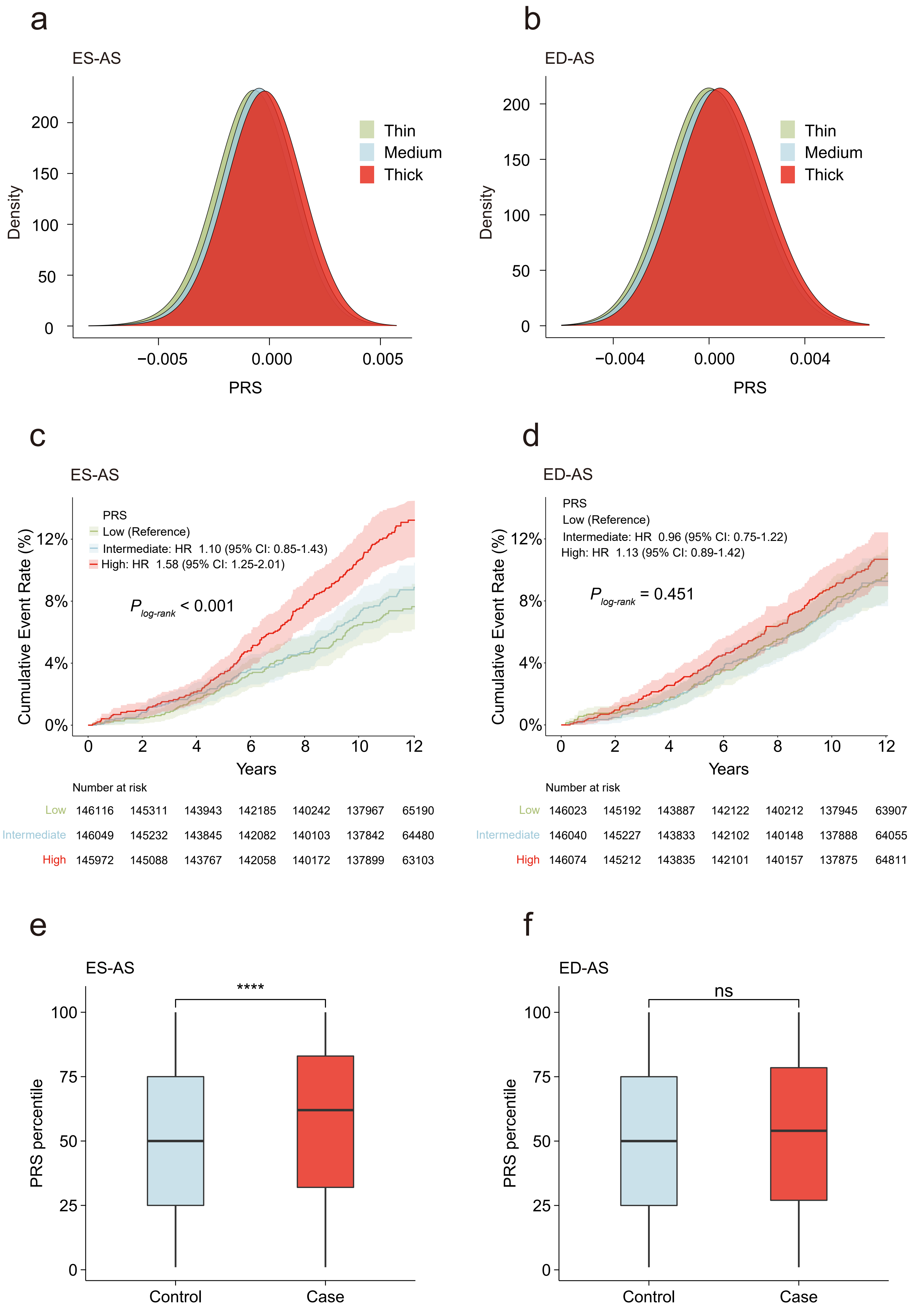

Supplementary Fig. 12. Distribution of PRS and cumulative incidence of HCM stratified by PRS of anterospetal LVRWT.

a-b. Distribution of PRS for participants with thin, medium and thick LVRWT. PRS of anterospetal LVRWT end systole (**a**) and end diastole (**b**) yielded discrimination for the LVRWTs. **c-d.** A total of 439,981 individuals unrelated to the CMR cohort. Those in the first tertiles of genetically predicted anterospetal LVRWT are depicted in green, the second tertiles are depicted in blue and the last tertiles are depicted in red. The darker shades represent the central estimate of the cumulative incidence (defined as 1-the Kaplan-Meier survival estimate). The lighter shades represent the respective 95% CIs. The x axis depicts years since enrollment in the UKB; the y axis depicts cumulative incidence of HCM. Strata based on genetic prediction of anterospetal LVRWT at end systole (**c**) and end diastole (**d**). **e-f.** Distribution of ES-AS (**e**) and ED-AS (**f**) PRS percentiles for HCM cases (n = 420) and controls (n = 439,561). For all box plots: central line of each box, median; top and bottom edges of each box, first and third quartiles; whiskers extend 1.5× the interquartile range beyond box edges. *P*-values were calculated by a two-sided Student's t-test. Asterisk denotes statistically significant differences * *P* <0.05; ** *P* <0.01; *** *P* <0.001. Abbreviation: PRS, polygenic risk score; HCM, hypertrophic cardiomyopathy; LVRWT, LV regional wall thickness; ES, end systole; ED, end diastole; AS, anterospetal.

Supplementary Fig 13

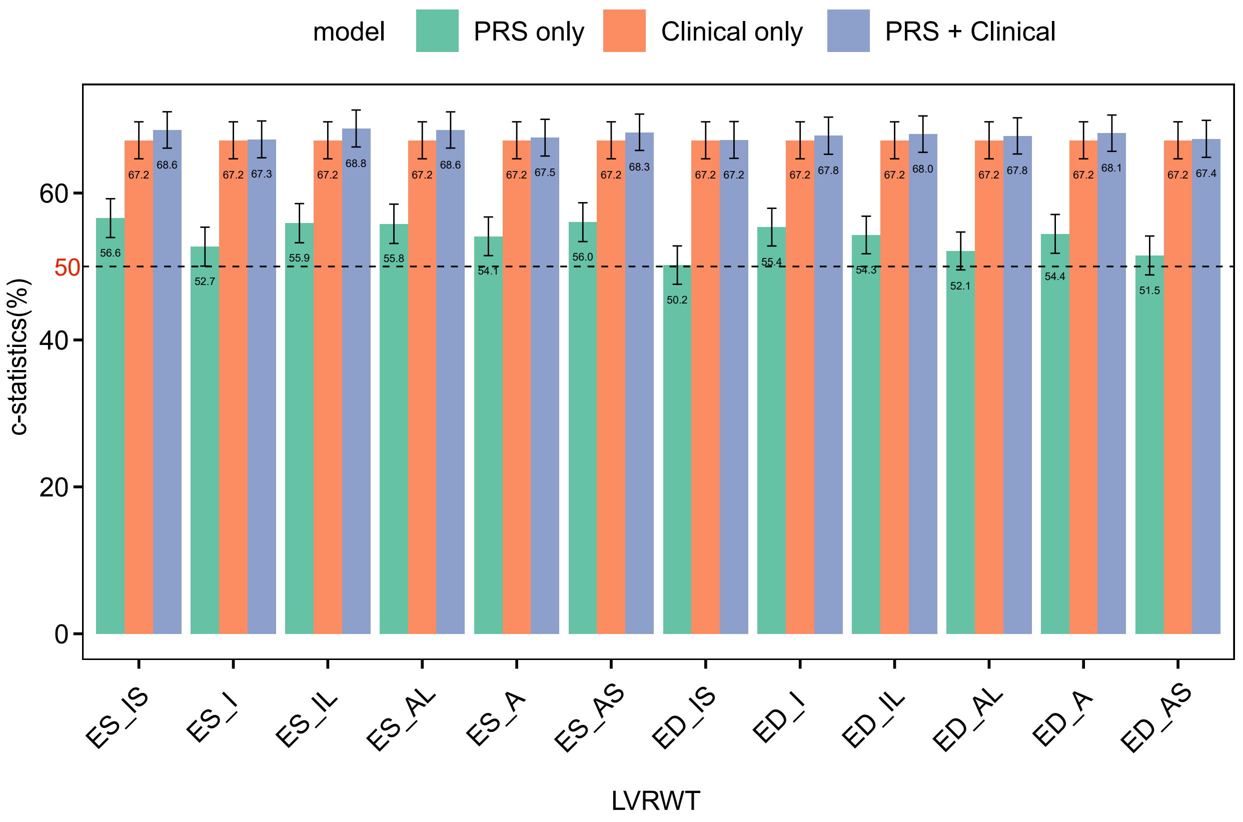

Supplementary Fig. 13. C-statstic results of 12 LVRWT PRSs for HCM prediction.
C-statistics are based on 11.8-year follow-up events from Cox regression models of listed variables. Clinical risk factors includes age , sex, BMI, smoking status, alcohol intake frequency and PC1-10 variables in its risk estimation. Abbreviation: PRS, polygenic risk score; IS, inferoseptal; I, inferior; IL, inferolateral; AL, anterolateral; A, anternor; AS, anterospetal; ES, end systole; ED, end diastole; LVRWT, LV regional wall thickness.

Supplementary Fig 14

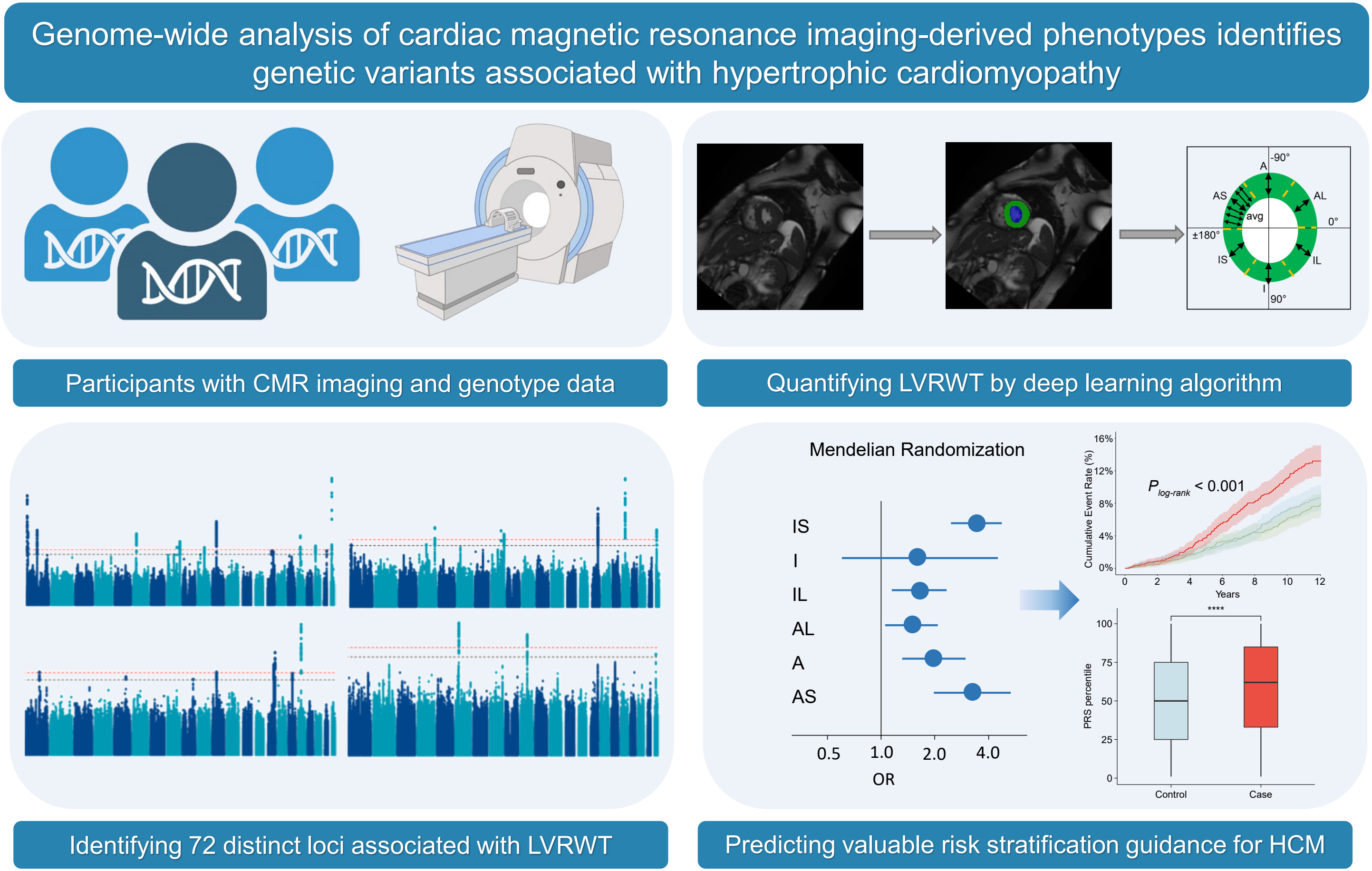

Supplementary Fig. 14. Graphical representation of GWAS on left ventricular regional wall thicknesses based on CMR imaging.

Deep learning algorithms were used to annotate 12 LVRWT using cardiac magnetic resonance imaging data from 42,194 European individuals. We identified 72 distinct loci associated with at least one LVRWT phenotype. Furthermore, using Mendelian randomization, we evaluated the causal relationships between the LVRWT traits and CVDs, and polygenic risk scores derived from LVRWT traits predicted incident HCM with favorable discrimination performance for high-risk HCM populations. Some elements in the figure were created using BioRender.com. Abbreviation: CMR, cardiac magnetic resonance; HCM, hypertrophic cardiomyopathy; LVRWT, LV regional wall thicknes; IS, inferoseptal; I, inferior; IL, inferolateral; AL, anterolateral; A, anternor; AS, anterospetal

Supplementary Fig 15

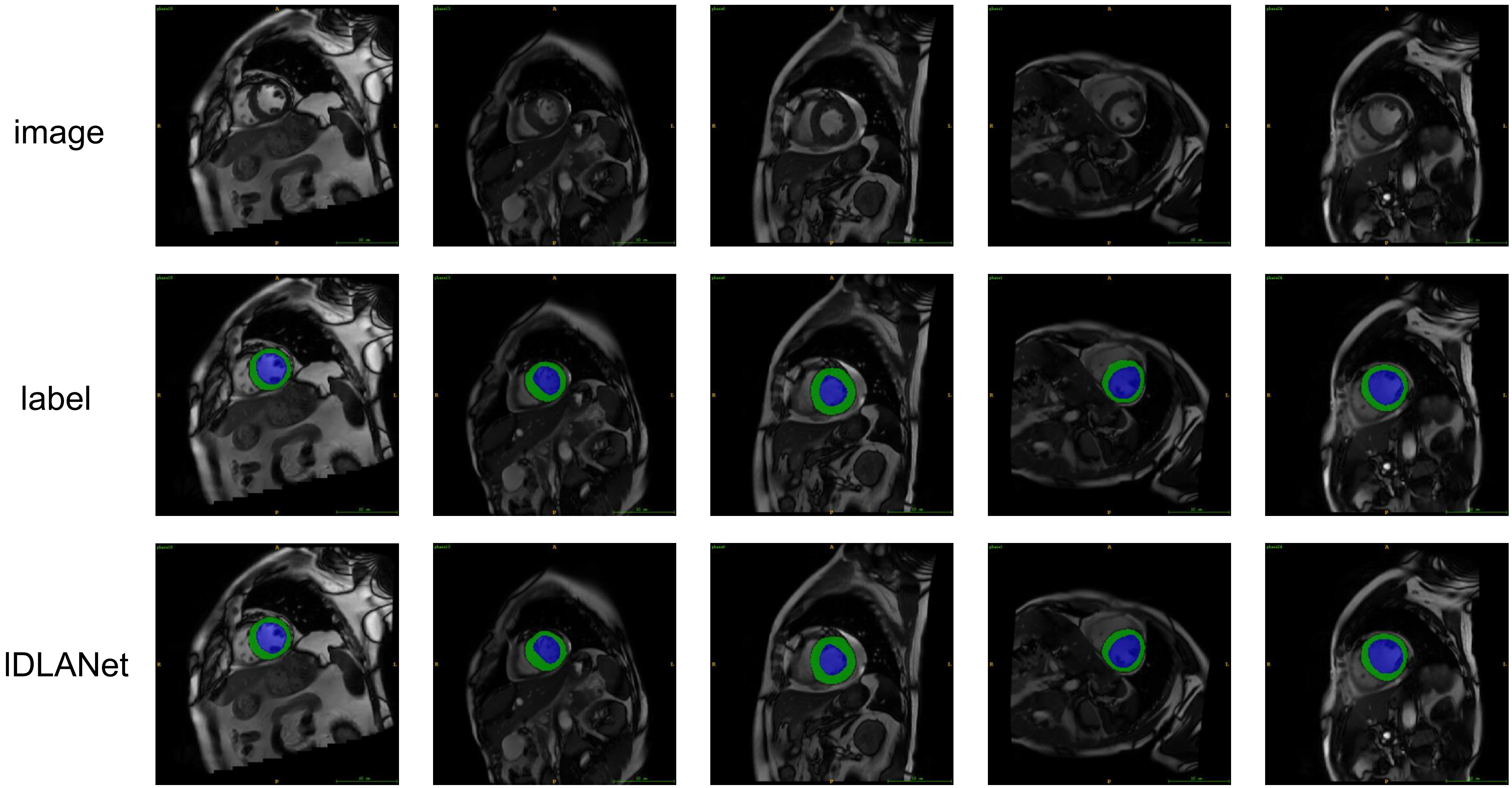

Supplementary Fig 15. Illustration of segmentation results of DLANet on the UKB dataset. First row: the original short axis images. Second row: manual labels by the doctors. Last row: the prediction results generated by the DLANet. Green: LV myocardium, blue: LV cavity

Supplementary Tables

Supplementary Table 1: Cohort characteristics of the UK Biobank

	Period	
**Characteristics**	**End systole**	**End diastole**
N_{max}	(N = 42,094)	(N = 42,194)
**Sex**		
Women	22175 (52.7)	22275 (52.8)
Men	19919 (47.3)	19919 (47.2)
**Age ,years**	64.1 (7.72)	64.1 (7.72)
**Body mass index,kg/m2**	26.2 (3.99)	26.2 (3.99)
**Smoking statues**		
Current	1406 (3.3)	1404 (3.3)
Previous	14078 (33.4)	14137 (33.5)
Never	26187 (62.2)	26231 (62.2)
Prefer not to answer	141 (0.3)	141 (0.3)
Unknown	282 (0.7)	281 (0.7)
**Drinking statues**		
Daily or almost daily	7164 (17.0)	7210 (17.1)
Three or four times a week	11883 (28.2)	11898 (28.2)
Once or twice a week	11002 (26.1)	11022 (26.1)
One to three times a month	4805 (11.4)	4810 (11.4)
Special occasions only	4214 (10.0)	4221 (10.0)
Prefer not to answer	2728 (6.5)	2736 (6.5)
Never	16 (0.0)	16 (0.0)
Unknown	282 (0.7)	281 (0.7)

Abbreviations: Numbers are mean (standard deviation) or total number (%); N_{max} represents the maximum number of every period

Supplementary Table 2: Cardiac magnetic resonance variables

	Period	
**Region**	**End systole**	**End diastole**
IS	11.7 (1.9)	7.8 (1.4)
I	11.5 (2.1)	7.1 (1.2)
IL	11.4 (2.1)	6.4 (1.0)
AL	11.0 (2.1)	6.2 (0.9)
A	10.3 (2.1)	6.1 (0.9)
AS	11.5 (1.9)	7.1 (1.2)

Numbers are mean (standard deviation); Abbreviations: IS, inferoseptal; I, infernor; IL,anterolateral; inferolateral;AL,anterolateral; A,anternor;AS,anterospetal

Supplementary References

1. Bernard, O. *et al.* Deep Learning Techniques for Automatic MRI Cardiac Multi-Structures Segmentation and Diagnosis: Is the Problem Solved? *IEEE Trans Med Imaging* **37**, 2514-2525 (2018).
2. Isensee, F., Jaeger, P.F., Kohl, S.A.A., Petersen, J. & Maier-Hein, K.H. nnU-Net: a self-configuring method for deep learning-based biomedical image segmentation. *Nat Methods* **18**, 203-211 (2021).
3. Dice, L.R.J.E. Measures of the amount of ecologic association between species. **26**, 297-302 (1945).
4. Sorensen, T.A.J.B.S. A method of establishing groups of equal amplitude in plant sociology based on similarity of species content and its application to analyses of the vegetation on Danish commons. **5**, 1-34 (1948).
5. Bai, W. *et al.* Automated cardiovascular magnetic resonance image analysis with fully convolutional networks. *J Cardiovasc Magn Reson* **20**, 65 (2018).
6. Wang, W. *et al.* Quantification of full left ventricular metrics via deep regression learning with contour-guidance. **7**, 47918-47928 (2019).
