# Supplementary material for: Genome-wide association analysis of left ventricular imaging-derived phenotypes identifies 72 risk loci and yields genetic insights into hypertrophic cardiomyopathy
Source: Nat Commun. 2023 Nov 30;14:7900. doi: 10.1038/s41467-023-43771-5 (PMC10689443; doi:10.1038/s41467-023-43771-5)
Supplement: Supplementary file 3 — Description of Additional Supplementary Files [file 41467_2023_43771_MOESM3_ESM.pdf]

Description of Additional Supplementary Files

Supplementary Data 1. Genomic loci identified for all LVRWT phenotypes

Supplementary Data 2. Data sources for genetic correlation and mendelian randomization analyses

Supplementary Data 3. eQTL significant gene

Supplementary Data 4. TWAS results for inferoseptal

Supplementary Data 5. TWAS results for inferior

Supplementary Data 6. TWAS results for inferolateral

Supplementary Data 7. TWAS results for anterolateral

Supplementary Data 8. TWAS results for anternor

Supplementary Data 9. TWAS results for anterospetal

Supplementary Data 10. MAGMA-defined genes at $P < 2.64 \times 10^{-6}$

Supplementary Data 11. summary of significant genes

Supplementary Data 12. Significantly enriched gene ontology (GO) terms

Supplementary Data 13. Tissue-specific enrichment

Supplementary Data 14. Genetic correlations between LVRWTs and cardiac function related traits

Supplementary Data 15. Genetic correlations between LVRWTs and heart disease

Supplementary Data 16. Causal effects between RWT and heart disease using Mendelian Randomization

Supplementary Data 17. polygenic score SNP weights

Supplementary Data 18. Associations between LVRWT PRS and incident disease

Supplementary Data 19. Disease definitions

Supplementary Data 20. Covariate definitions

Supplementary Data 21. Testing results on ACDC dataset with different methods for comparison

Supplementary Data 22. Testing results on UKB dataset with different methods for comparison

Supplementary Data 23. Testing results of DLANet on ACDC dataset by using different number of training data

Supplementary Data 24. Testing results of DLANet on UKB dataset by using different number of training data
