# Supplementary material for: Genome-wide association analysis of left ventricular imaging-derived phenotypes identifies 72 risk loci and yields genetic insights into hypertrophic cardiomyopathy
Source: Nat Commun. 2023 Nov 30;14:7900. doi: 10.1038/s41467-023-43771-5 (PMC10689443; doi:10.1038/s41467-023-43771-5)
Supplement: Supplementary file 28 — Reporting Summary [file 41467_2023_43771_MOESM28_ESM.pdf]

nature portfolio

Corresponding author(s): Shaoting Zhang, Jianbo Tian & Xiaoping Miao

Last updated by author(s): Nov 3, 2023

Reporting Summary

Nature Portfolio wishes to improve the reproducibility of the work that we publish. This form provides structure for consistency and transparency in reporting. For further information on Nature Portfolio policies, see our Editorial Policies and the Editorial Policy Checklist.

Statistics

For all statistical analyses, confirm that the following items are present in the figure legend, table legend, main text, or Methods section.

n/a	Confirmed	
☐	☒	The exact sample size (*n*) for each experimental group/condition, given as a discrete number and unit of measurement
☐	☒	A statement on whether measurements were taken from distinct samples or whether the same sample was measured repeatedly
☐	☒	The statistical test(s) used AND whether they are one- or two-sided<br>*Only common tests should be described solely by name; describe more complex techniques in the Methods section.*
☐	☒	A description of all covariates tested
☐	☒	A description of any assumptions or corrections, such as tests of normality and adjustment for multiple comparisons
☐	☒	A full description of the statistical parameters including central tendency (e.g. means) or other basic estimates (e.g. regression coefficient) AND variation (e.g. standard deviation) or associated estimates of uncertainty (e.g. confidence intervals)
☐	☒	For null hypothesis testing, the test statistic (e.g. *F*, *t*, *r*) with confidence intervals, effect sizes, degrees of freedom and *P* value noted<br>*Give P values as exact values whenever suitable.*
☒	☐	For Bayesian analysis, information on the choice of priors and Markov chain Monte Carlo settings
☐	☒	For hierarchical and complex designs, identification of the appropriate level for tests and full reporting of outcomes
☐	☒	Estimates of effect sizes (e.g. Cohen's *d*, Pearson's *r*), indicating how they were calculated

Our web collection on statistics for biologists contains articles on many of the points above.

Software and code

Policy information about availability of computer code

Data collection	To segments and quantificate myocardial wall thicknesses, we established a novel deep-learning framework named Myocardial Segmentation and Measurement Method (MSMM).
Data analysis	-pytorch v1.9.1<br>-BOLT v2.3.6<br>-plink 1.9<br>-snpeff 5.1<br>-bedtools 2.29.1<br>-R4.1<br>-ldsc 1.0.1<br>-FUSION-TWAS (http://gusevlab.org/projects/fusion/#installation)

For manuscripts utilizing custom algorithms or software that are central to the research but not yet described in published literature, software must be made available to editors and reviewers. We strongly encourage code deposition in a community repository (e.g. GitHub). See the Nature Portfolio guidelines for submitting code & software for further information.

Data

Policy information about availability of data

All manuscripts must include a data availability statement. This statement should provide the following information, where applicable:

- Accession codes, unique identifiers, or web links for publicly available datasets
- A description of any restrictions on data availability
- For clinical datasets or third party data, please ensure that the statement adheres to our policy

-All cardiovascular measurements that we performed in the UK Biobank will be returned to the UK Biobank within 6 months of publication for future use by approved UK Biobank researchers.
-UK Biobank data are made available to researchers from research institutions with genuine research inquiries, following UK Biobank approval by requesting access at https://bbams. ndph. ox. ac. uk/ams/signup
All other data are contained within the article and its supplementary information.

Research involving human participants, their data, or biological material

Policy information about studies with human participants or human data. See also policy information about sex, gender (identity/presentation), and sexual orientation and race, ethnicity and racism.

Reporting on sex and gender	We took sex into considerations in our study and our findings could apply to both male and female. Sex in the UK Biobank was determined based on self-reporting data via questionnaire, and all included participants gave written informed consent for sharing of individual-level data.
Reporting on race, ethnicity, or other socially relevant groupings	In our study, the study population primarily consisted of individuals of European descent, with race defined based on self-reporting data collected through questionnaires. The Genome-Wide Association Study (GWAS) analysis in our research was conducted using the BOLT-LMM software. We adjusted for various covariates, including age, sex, body mass index (BMI), and the first ten principal components (PC1-PC10).BOLT-LMM was chosen for its ability to account for ancestral heterogeneity, cryptic population structure, and sample relatedness. This is achieved through the application of a linear mixed model with a Bayesian mixture prior serving as a random effect in the analysis.In addition, we conducted a population stratification assessment using the ldsc software. The results of this analysis indicated no significant population substructure, and more detailed information can be found in the 'Results' section of our study.
Population characteristics	Comprehensive details regarding the population characteristics can be found in Supplementary Table 1 of the manuscript. The study cohort primarily consisted of individuals with European ancestry. During the first imaging visit, the average age of the participants was approximately 64.1 years. Notably, about 47.3% of the cohort was male at both the end systole and end diastole time points. Additionally, roughly 3.3% of participants reported current tobacco smoking at both end systole and end diastole, while 17.1% indicated daily alcohol consumption at end systole, and 17.0% reported daily alcohol consumption at end diastole.
Recruitment	The study authors had no contact with any participant. As described by the UK Biobank investigators, individuals aged 40-69 in the UK were recruited via mailer from 2006-2010. Participants chosen to undergo magnetic resonance imaging in the UK Biobank are reported to have been chosen due to proximity to imaging centers, and otherwise at random. Several biases arise from this. The study population largely consisted of European-ancestry UK Biobank participants, limiting generalizability to other populations. In addition, volunteer-based biobanks such as the UK Biobank can differ from the general population by largely being healthier and more female (healthy volunteer bias). There is selection in terms of the requirement for survival to middle-age in order to enroll in the UK Biobank, screening out individuals with severe disease that would cause death in early life or childhood. Finally, individuals had to survive for additional time after enrollment in the UK Biobank in order to undergo MRI(i.e., MRI was not performed upon enrollment). All of these factors enrich the study population for people who are healthier than a general population.
Ethics oversight	Our analyses of UK Biobank data were approved by the Institutional Review Board of Wuhan university (ID:2022042).

Note that full information on the approval of the study protocol must also be provided in the manuscript.

Field-specific reporting

Please select the one below that is the best fit for your research. If you are not sure, read the appropriate sections before making your selection.

☒ Life sciences ☐ Behavioural & social sciences ☐ Ecological, evolutionary & environmental sciences

For a reference copy of the document with all sections, see nature.com/documents/nr-reporting-summary-flat.pdf

Life sciences study design

All studies must disclose on these points even when the disclosure is negative.

Sample size	45,353 Uk Biobank participants had cardiac MRI data available at the time of the study. This sample size was determined by using the complete amount of data made available by the Uk Biobank at the time of analysis.

Data exclusions	Please see Supplementary Figure 1 for more detail. In brief, we excluding participants with poor imaging, missing information on genetic data, previous myocardial infarction, diagnosis of heart failure as well as body mass index (BMI) < 16 or > 40 kg/m2. Finally, 42,194 individuals were included in our analysis.
Replication	The discovery GWAS signals were replicated in the remaining 439,981 individuals without CMR imaging data of UK Biobank.
Randomization	Given the observational nature of this study and GWAS design, randomization was not applicable.
Blinding	CMR image analysts were blinded to participants' genotype, demographic and clinical characteristics.

Reporting for specific materials, systems and methods

We require information from authors about some types of materials, experimental systems and methods used in many studies. Here, indicate whether each material, system or method listed is relevant to your study. If you are not sure if a list item applies to your research, read the appropriate section before selecting a response.

Materials & experimental systems

n/a	Involved in the study	
☒	☐	Antibodies
☒	☐	Eukaryotic cell lines
☒	☐	Palaeontology and archaeology
☒	☐	Animals and other organisms
☒	☐	Clinical data
☒	☐	Dual use research of concern
☒	☐	Plants

Methods

n/a	Involved in the study	
☒	☐	ChIP-seq
☒	☐	Flow cytometry
☒	☐	MRI-based neuroimaging

Plants

Seed stocks	We conducted research on the human body, and it does not involve plants.
Novel plant genotypes	We conducted research on the human body, and it does not involve plants.
Authentication	We conducted research on the human body, and it does not involve plants.
